# Deciphering the Genetic Architecture of Plant Height in Soybean Using Two RIL Populations Sharing a Common *M8206* Parent

**DOI:** 10.3390/plants8100373

**Published:** 2019-09-26

**Authors:** Yongce Cao, Shuguang Li, Guoliang Chen, Yanfeng Wang, Javaid Akhter Bhat, Benjamin Karikari, Jiejie Kong, Junyi Gai, Tuanjie Zhao

**Affiliations:** 1 MOA Key Laboratory of Biology and Genetic Improvement of Soybean (General), State Key Laboratory for Crop Genetics and Germplasm Enhancement, Soybean Research Institute, National Center for Soybean Improvement, Nanjing Agricultural University, Nanjing 210095, China; caoyongce@yau.edu.cn (Y.C.); dawn0524@126.com (S.L.); javid.akhter69@gmail.com (J.A.B.); benkarikari1@gmail.com (B.K.); 2012094@njau.edu.cn (J.K.); 2 Shaanxi Key Laboratory of Chinese Jujube, College of Life Science, Yan’an University, Yan’an 716000, China; glc@yau.edu.cn (G.C.); yadxwyf@yau.edu.cn (Y.W.)

**Keywords:** linkage mapping, sub-populations, high-density bin map, main-effect QTL, interaction effects

## Abstract

Plant height (PH) is an important agronomic trait that is closely related to soybean yield and quality. However, it is a complex quantitative trait governed by multiple genes and is influenced by environment. Unraveling the genetic mechanism involved in PH, and developing soybean cultivars with desirable PH is an imperative goal for soybean breeding. In this regard, the present study used high-density linkage maps of two related recombinant inbred line (RIL) populations viz., MT and ZM evaluated in three different environments to detect additive and epistatic effect quantitative trait loci (QTLs) as well as their interaction with environments for PH in Chinese summer planting soybean. A total of eight and 12 QTLs were detected by combining the composite interval mapping (CIM) and mixed-model based composite interval mapping (MCIM) methods in MT and ZM populations, respectively. Among these QTLs, nine QTLs viz., *QPH-2*, *qPH-6-2_MT_*, *QPH-6*, *qPH-9-1_ZM_*, *qPH-10-1_ZM_*, *qPH-13-1_ZM_*, *qPH-16-1_MT_*, *QPH-17* and *QPH-19* were consistently identified in multiple environments or populations, hence were regarded as stable QTLs. Furthermore, Out of these QTLs, three QTLs viz., *qPH-4-2_ZM_*, *qPH-15-1_MT_* and *QPH-17* were novel. In particular, *QPH-17* could detect in both populations, which was also considered as a stable and major QTL in Chinese summer planting soybean. Moreover, eleven QTLs revealed significant additive effects in both populations, and out of them only six showed additive by environment interaction effects, and the environment-independent QTLs showed higher additive effects. Finally, six digenic epistatic QTLs pairs were identified and only four additive effect QTLs viz., *qPH-6-2_MT_*, *qPH-19-1_MT_*/*QPH-19*, *qPH-5-1_ZM_* and *qPH-17-1_ZM_* showed epistatic effects. These results indicate that environment and epistatic interaction effects have significant influence in determining genetic basis of PH in soybean. These results would not only increase our understanding of the genetic control of plant height in summer planting soybean but also provide support for implementing marker assisted selection (MAS) in developing cultivars with ideal plant height as well as gene cloning to elucidate the mechanisms of plant height.

## 1. Introduction

Soybean (*Glycine max*) is one of the most important crops grown throughout the world, and is a primary source of plant oil and protein for the human diet [1]. In China, the soybean yield increases slowly in the past 50 years. Moreover, China imports more than 80% of the soybeans needed for the total domestic use, hence it is priority to increase the domestic production of soybean to make country self-sufficient [2]. In this context, development of high yielding soybean cultivars is the key objective in ongoing plant breeding programmes. There is a positive correlation between plant height (PH) and pod number per plant, but taller plants are also more prone to lodging, which affects seed yield and quality. However, PH is an important agronomic trait that is closely related with seed yield in soybean [3,4], thereby making PH an important trait in soybean breeding program. However, PH is a complex quantitative trait controlled by multiple major or minor genes/QTLs, and is highly affected by the environment and other interactions [5,6]. Although, both conventional breeding and marker-assisted selection (MAS) have been successfully used to breed for PH. The latter is a more effective breeding method especially for environment-sensitive traits, and can be used to select plants at earlier seedling stages for those traits that are expressed late in the maturity stage [7,8]. Therefore, unrevealing the genetic basis of PH will greatly facilitates the implementation of MAS in breeding high-yielding soybean varieties.

In the past three decades, numerous studies have been carried out for elucidating the genetic control and QTLs composition of PH in soybean [9,10,11,12]. Till date, more than 200 QTLs were documented in SoyBase [13], that are distributed on all 20 chromosomes of soybean, and have been detected in different populations and environments. Due to lack of high-throughput markers, the genetic maps used in most of the previous studies for QTL detection were based on low-throughput markers, such as restriction fragment length polymorphism (RFLP), amplified fragment length polymorphism (AFLP) and simple sequence repeat (SSR). As a result, most of these QTLs were mapped in large genomic regions, and have not been effectively used in MAS to breed varieties with ideal PH. Compared to low-throughput marker, the single nucleotide polymorphisms (SNPs) are the most abundant genetic polymorphism present in the plant genome and have high density as well as are evenly distributed across the whole genome. With the rapid advances in sequencing technologies and the completion of the whole genome sequencing of soybean *cv. Williams 82* [14], SNP markers have become the marker of choice and has been now routinely used for high-density genetic map construction in soybean [15]. These high-density linkage maps have greatly assisted in increasing QTL mapping resolution as well as provided convenience for QTL fine mapping and MAS for complex traits. For example, Li et al. [16] used a soybean genetic map with an average distance of 0.72 cM between adjacent markers to detect QTLs for fatty acids and the results shown that more than 90% of QTL intervals were smaller than 5.0 cM. Similarly, Zhang et al. [17] reported that the confidence interval of QTLs for tolerance to low-phosphorus stress in soybean could be significantly reduced by using a high-density genetic map.

Although some of the quantitative traits are controlled by only few major QTLs/genes, while the phenotypic variation for most of the complex traits is governed by many factors [18]. In addition, two important factors i.e., QTL by QTL (epistatic) and QTL by environments interactions, contribute significantly to phenotypic variation of a complex trait [19]. When the additive and epistatic effects as well as QTL by environment interaction effects associated with the target trait are reckoned in the QTL mapping model, the precision of QTL mapping would be greatly improved [20]. Therefore, these factors cannot only be considered as the main obstacles to dissect the genetic architecture of complex traits, but also affect the accuracy of breeding value estimation and thus hindering the efficiency of breeding programs. Hence, these factors should be considered while dissecting the genetic basis of complex traits, and their use to improve plant performance. In recent years, epistatic and QTL by environment interaction effects have been considered in several crop species including soybean for QTL mapping [3,21,22,23,24]. However, to our knowledge most of the previous studies on QTL mapping for PH in soybean focused mostly main-effect QTLs mapping [25,26,27]. Therefore, efforts are required to study such QTL interaction effects for precise breeding of ideal PH in soybean.

In addition to the minor effect QTLs, PH was reported to be regulated by two major growth habit genes, *Dt1* and *Dt2* [28,29,30]. In the Chinese Jang-Huai River Valley, soybean is always planted in early June and harvested at early October. In this region, soybean varieties have both determinate and indeterminate stem growth habits.

However, very little is known about the genetics of PH in the summer planting soybean genotypes grown in this region. By keeping the above into consideration, the present study has used three parents, *M8206* (M), *Tongshan* (T) and *Zhengyang* (Z) with different growth habits to develop two related RIL populations viz., MT and ZM sharing a common indeterminate parent *M8206*. The objective of our study was to estimate the main-additive effects, epistatic effects and QTL by environment interaction effects of QTLs by utilizing the high-density linkage maps as well as phenotypic data collected from three different environments of two RIL populations to get detailed understanding of genetic basis for PH in summer planting soybean. This will further assist to better understand the implications of these QTLs while breeding for ideal PH through MAS method.

## 2. Results

### 2.1. Evaluation of Phenotypic Variation for Plant Height

**Figure 1:**
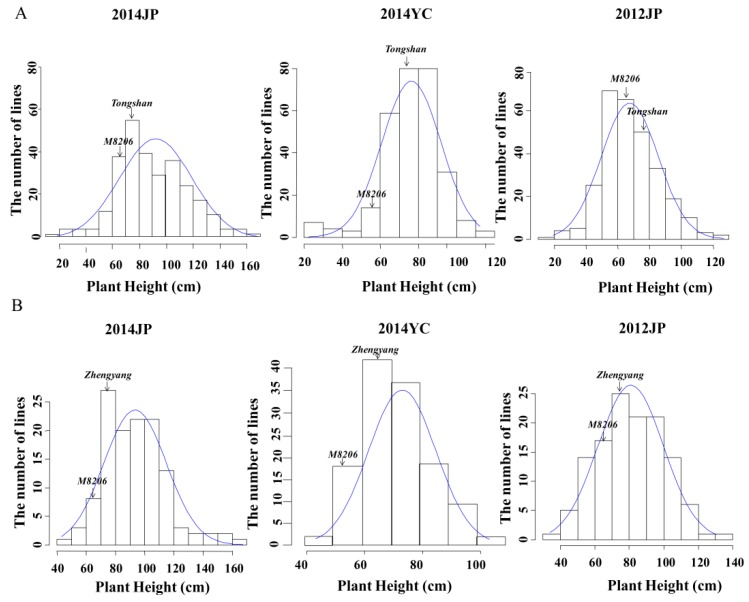
Frequency distribution of plant height in three environments. (**A**) MT population; (**B**) ZM population. Where JP and YC represents Jiangpu experiment station and Yancheng experiment station, respectively.

Phenotypic performance of PH among two RIL populations viz., MT and ZM along with their parents across three different environments (2012JP, 2014JP and 2014YC, where 2012 and 2014 represent the test year, JP and YC represent the test site.) are presented in Table 1 and Figure 1. The average PH between the two parents in both RIL populations across all environments were significantly different (*p <* 0.05). Plant height of determinate parents viz., *Tongshan* and *Zhengyang* were in average of 73.3 – 75.4 cm and 75.7 – 77.7 cm, respectively and were higher than that of the common parent *M8206* across different environments (Table 1). Mean value of many RILs exceeded their parents in both directions for MT and ZM mapping populations indicating transgressive segregation of RILs for PH (Figure 1).

Moreover, in all three studied environments absolute values of skewness and kurtosis of PH in both MT and ZM populations were < 1 across all three environments except the kurtosis value of MT population in the 2014YC environment (Table 1). These result indicates that the segregations of PH follows normal distribution in both RIL populations, and indicates that PH is controlled by multiple genes, and are thus suitable for QTL mapping. The phenotypic values of PH exhibited wide range, with the coefficient of variation (CV) ranging from 20.4 to 29.5% in MT population, and 16.0 to 23.5% in ZM population (Table 1).

Estimates of broad-sense of heritability (*h*^2^) were high (> 90%) for PH in both populations (Table 1), suggesting that considerable proportion of phenotypic variation of PH is under genetic control. Analysis of variance (ANOVA) revealed that genotype (G), environmental (E) and genotype by environment (G × E) interaction effects were significantly high (*p <* 0.01) for PH in both RIL populations, and mean square (MS) value for the G × E interaction was less than that of the genotype (Table S1).

### 2.2. QTL Mapping for Plant Height by CIM Method in MT and ZM Populations

**Figure 2:**
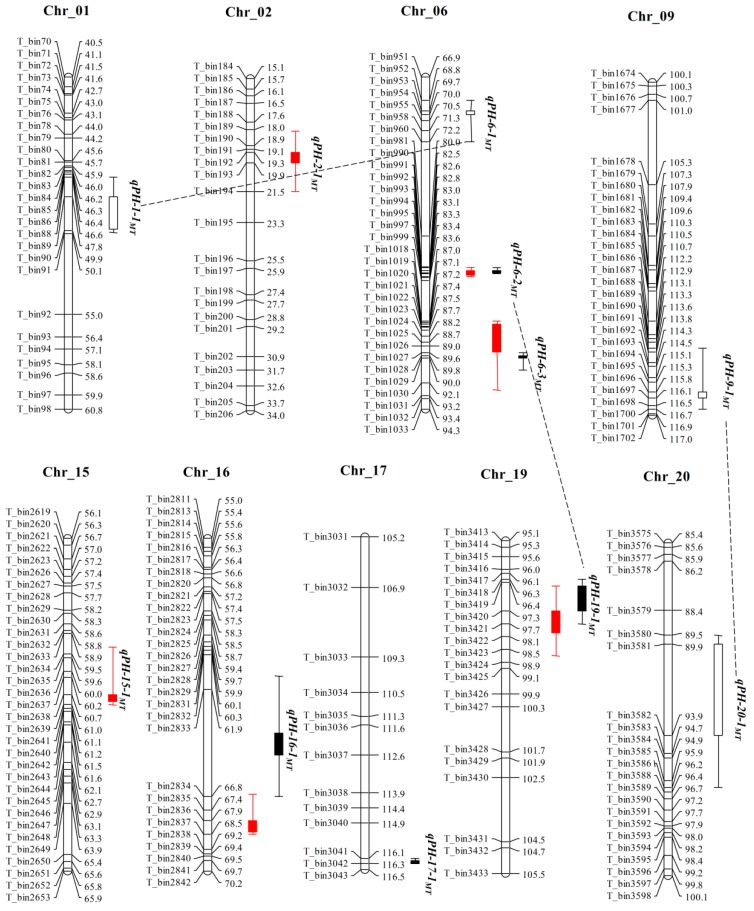
Distribution of QTLs on genetic map for plant height in the MT population (here only the genetic map around QTLs is shown). Red graphics represent the QTLs were detected by CIM method. Black graphics represent the QTLs were detected by MCIM method, and the filled graphics represent QTLs with additive effect, while the hollow graphics represent QTLs with non-additive effect. Black dotted lines represent that there are epistatic interaction effects between two connected QTLs.

**Figure 3:**
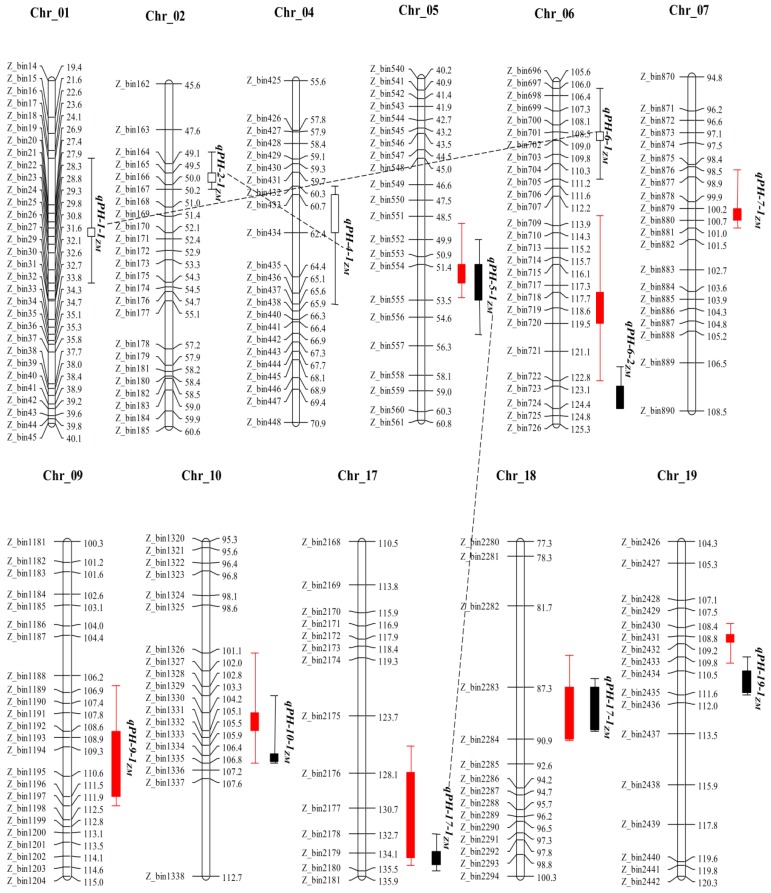
Distribution of QTLs on genetic map for plant height in the ZM population (here only the genetic map around QTLs is shown). Red graphics represent the QTLs were detected by CIM method. Black graphics represent the QTLs were detected by MCIM method, and the filled graphics represent QTLs with additive effect, while the hollow graphics represent QTLs with non-additive effect. Black dotted lines represent that there are epistatic interaction effects between two connected QTLs.

Linkage analysis were performed using the high-density genetic maps of MT and ZM populations for the identification of QTLs for PH in soybean, and the QTLs identified by composite interval mapping (CIM) are shown in Table 2, Figure 2 and Figure 3. In total, 14 QTLs explaining 1.8 to 50.7% of the phenotypic variation (*R*^2^) associated with PH were detected in two RIL populations in three different environments. Two QTL pairs viz., *qPH-6-3_MT_* & *qPH-6-2_ZM_* and *qPH-19-1_MT_* & *qPH-19-1_ZM_* were detected in same physical genomic position on Chr6 and Chr19 in MT and ZM population, respectively. Each pair was considered as same QTL, and renamed as *QPH-6* and *QPH-19*. These QTLs (*QPH-6* and *QPH-19*) had *R*^2^ > 10% in both populations and each environment. Therefore, these two QTLs are considered as the most stable major QTLs in this study. These QTLs regions will be the main targets for fine mapping, candidate gene identification and marker-assisted breeding of PH in summer planting soybean.

For the MT population, a total of six QTLs distributed on five chromosomes (Chr2, Chr6, Chr15, Chr16 and Chr19) were identified (in at least one environment) with LOD scores ranging from 3.2 to 55.7, and explained 1.8 to 50.7% of the phenotypic variation (Table 2 and Figure 2). Among them, *qPH-6-2_MT_* and *qPH-19-1_MT_*/*QPH-19* can be considered as environmentally stable QTLs, which were detected in all environments and could explain 13.7 to 19.4% and 22.2 to 50.7% of the phenotypic variation, respectively. *qPH-6-3_MT_*/*QPH-6* was another important QTL that was detected in two environments and explained an average of 16.1% of the phenotypic variation. The other three QTLs were detected in a single environment. The positive alleles of *qPH-2-1_MT_*, *qPH-6-2_MT_*, *qPH-6-3_MT_*/*QPH-6* and *qPH-16-1_MT_* came from *Tongshan*, while others came from *M8206* (Table 2). Out of six QTLs detected in MT population, *qPH-15-1_MT_* was identified for the first time, however the remaining five QTLs were previously reported but were located in small genomic regions in the present study, that might provide more detailed information for gene identification (Table 2).

For the ZM population, a total of eight QTLs distributed on eight different chromosomes (Chr5, Chr6, Chr7, Chr9, Chr10, Chr17, Chr18 & Chr19) were identified for PH with LOD score ranged from 2.7 to 15.0, and explained 3.9 to 30.9% of the phenotypic variation in different individual environments (Table 2 and Figure 3). Among them, *qPH-17-1_ZM_* was the most stable QTL detected in all the three environments and could explain 4.2 to 9.0% of the phenotypic variation. *qPH-6-2_ZM_*/*QPH-6* and *qPH-19-1_ZM_*/*QPH-19* were detected in two environments and explained 11.3–12.7% and 19.7 to 30.9% of the phenotypic variation, respectively. These QTLs are major QTLs for PH in ZM population. *qPH-9-1_ZM_* and *qPH-10-1_ZM_* were also detected in two environments with each accounting for less than 10% of the phenotypic variation. *qPH-5-1_ZM_*, *qPH-7-1_ZM_* and *qPH-18-1_ZM_* were detected in a single environment. The positive alleles of *qPH-5-1_ZM_*, *qPH-6-2_ZM_*, *qPH-9-1_ZM_* and *qPH-17-1_ZM_* came from *Zhengyang*, while others came from *M8206*. Out of eight QTLs detected in ZM population, only *qPH-17-1_ZM_* was detected for the first time, and the remaining seven QTLs were previously reported but were detected in small genomic regions in this study (Table 2).

### 2.3. Additive Effect QTLs and QTL by Environment Interaction Analysis

To further validate the QTLs detected by CIM, we performed another method of mixed-model based composite interval mapping (MCIM) to dissect the additive effect QTLs and QTL by environment interaction. By using MCIM method we identified a total of five additive effect QTLs for PH distributed on four chromosomes, and explained 1.7 to 39.3% of the phenotypic variation in MT population (Table 3). Interestingly, *qPH-6-2_MT_* and *qPH-19-1_MT_/QPH-19* were considered as major QTLs that had largest additive effects of 7.5 and 12.9 cm, and explained 13.5% and 39.3% of the phenotypic variation, respectively. Other three QTLs viz., *qPH-6-3_MT_*, *qPH-16-1_MT_* and *qPH-17-1_MT_/QPH-17* were minor with *R*^2^ < 10% (Table 3).

Among these five QTLs, two QTLs viz., *qPH-6-2_MT_* and *qPH-19-1_MT_/QPH-19* showed significant additive by environment interaction (AE) effects. However, *qPH-19-1_MT_/QPH-19* revealed AE effect at all environments, while as *qPH-6-2_MT_* had AE effect in two specific environments. The AE effects of *qPH-19-1_MT_/QPH-19* and *qPH-6-1_MT_* for PH could explain 6.9% and 1.9% of the phenotypic variation, respectively. In addition, the additive effect of *qPH-19-1_MT_/QPH-19* was positive, indicating that the positive alleles came from *M8206*. In contrast, the positive alleles of *qPH-6-2_MT_*, *qPH-6-3_MT_*, *qPH-16-1_MT_*, and *qPH-17-1_MT_/QPH-17* were inherited from *Tongshan*. Out of five additive effect QTLs identified in MT population, only *qPH-17-1_MT_/QPH-17* was novel. 

In the ZM population, a total of six additive effect QTLs distributed on six chromosomes (Chr5, Chr6, Chr10, Chr17, Chr19 and Chr19) were identified by MCIM method (Table 3). The additive effect of these QTLs varied from −2.5 to 6.3 cm, and explained 2.1 to 12.5% of the phenotypic variation. Among these six QTLs, *qPH-19-1_ZM_/QPH19* was the major QTL with the largest additive effect QTL (6.3 cm) and explained 12.5% of the phenotypic variation, which also had significantly high AE interaction in all environments, and the AE effect could explain 4.5% of the phenotypic variation. Furthermore, *qPH-6-2_ZM_*, *qPH-10-1_ZM_* and *qPH-17-1_ZM_* showed significant AE effect only in one specific environment, and explained 0.9%, 1.0% and 0.7% of the phenotypic variation, respectively. The *qPH-5-1_ZM_* and *qPH-18-1_ZM_* exhibited only additive effects with no AE interaction effects. The positive alleles of *qPH-5-1_ZM_*, *qPH-6-2_ZM_* and *qPH-17-1_ZM_* came from *Zhengyang*, while as positive alleles of remaining QTLs are derived from *M8206*. Out of six additive-effect QTLs identified in ZM population, only *qPH-17-1_ZM_ /QPH-17* was novel. 

Lastly, we performed a comparative analysis of QTLs detected by CIM and MCIM. A total of 14 and 11 QTLs were identified by CIM and MCIM, respectively. Among these QTLs, nine QTLs were common detected by both methods, indicating that these QTLs were stable and reliable. In addition, by comparing the physical genomic regions of QTLs identified in both populations (MT and ZM) and mapping methods (CIM and MCIM), a total of two QTLs viz., qPH-15-1_MT_ and QPH-17 were identified for the first time and are considered as novel QTLs identified in the present study (Table 2 and Table 3). The remaining previously reported QTLs were detected in small genomic regions. It is noteworthy that three genomic regions/QTLs viz., QPH-6, QPH-17 and QPH-19 located on Chr6, Chr17 and Ch19 were identified in both populations. Hence, these QTLs were considered as most stable QTLs that can be considered as the potential candidate genomic regions for breeding PH in summer planting soybean.

### 2.4. Epistatic Interaction Effects

A total of three pairs of epistatic QTL were identified for PH in MT populations (Table 4 and Figure 2). The Pair-1 consists of two additive QTLs, *qPH-19-1_MT_*/*QPH-19* and *qPH-6-2_MT_*/*QPH-6* located on Chr19 and Chr6, respectively. This epistatic QTL pair has both significant AA and AAE interaction effects, and explained 0.3% and 1.6% of the phenotypic variation, respectively (Table 4). The other two pairs epistatic QTL pairs viz., *qPH-1-1_MT_* & *qPH-6-1_MT_* and *qPH-9-1_MT_* & *qPH-20-1_MT_* had only AA epistatic effects, and explained 1.6% and 1.6% of the phenotypic variation, respectively.

In the ZM population, there are also three pairs of epistatic QTL identified (Table 4 and Figure 3). One pair consisted of two additive QTLs (*qPH-5-1_ZM_* and *qPH-17-1_ZM_*/*QPH-17*), and explained 1.6% of the phenotypic variation, and another pair consisted of two non-additive QTLs (*qPH-1-1_ZM_* and *qPH-6-1_ZM_*) and explained 1.5% of the phenotypic variation. Both these epistatic QTL pairs do not possess significant AAE interaction effects. However, the third QTL pair comprised two non-additive QTLs (*qPH-2-2_ZM_* and *qPH-4-1_ZM_*), and this pair of epistatic QTLs had both significant AA and AAE interaction effects, and explained 3.4% and 1.1% of the phenotypic variation, respectively. Hence, the above findings indicate that environment and epistatic interaction effects has considerable influence on the regulation of phenotypic expression of PH in summer planting soybean. However, except four QTLs viz., *qPH-6-2_MT_*, *qPH-19-1_MT_*, *qPH-5-1_ZM_* and *qPH-17-1_ZM_*, all the remaining additive effect QTLs did not show any epistatic effects.

### 2.5. QTLs Mapping in Subpopulations of MT and ZM Populations

Because the *Dt1* gene has a great effect on soybean plant height, in order to exclude the influence of *Dt1* locus on QTL mapping, the MT and ZM populations were divided into the determinate stem growth habit (MT-D and ZM-D) sub-population and the indeterminate stem growth habit (MT-I and ZM-I) sub-population according to the genotype of each line at *Dt1* locus. Then, the two sub-populations of MT and ZM populations were used to detect the QTLs that independent of the *Dt1* locus. And the QTLs identified in each sub-population by CIM method are shown in Table 5.

A total of two and four QTLs for plant height were identified by CIM method in MT-D and MT-I sub-populations, respectively (Table 5). One QTL, *qPH-6-2_MT_* could be detected in two sub-populations. In MT-D sub-population, two QTLs, *qPH-6-2_MT_* and *qPH-16-1_MT_* were detected in all environments, and could explain 16.7 to 49.6% and 6.2 to 7.4% of the phenotypic variation, respectively, which can be considered as environmentally stable QTLs. In MT-I sub-population, a total of four QTLs were identified. Their LOD values ranged from 3.4 to 15.8, which explained 5.9 to 29.2% of the phenotypic variation of individual QTL. Among them, *qPH-6-3_MT_*/*QPH-6* was the most stable QTL detected in all three environments and could explain 18.3 to 29.2% of phenotypic variation. *qPH-6-2_MT_* was detected in two environments and explained 21.4 to 28.8% of the phenotypic variation could also consider as a major QTL for PH in MT-I sub-population. *qPH-15-2_MT_* and *qPH-17-1_MT_*/*QPH-17* were detected in a single environment. The positive alleles of *qPH-15-2_MT_* came from *M8206*, while the other five QTLs came from *Tongshan.*

A total of three and four QTLs for plant height were identified by CIM method in ZM-I and ZM-D sub-populations, respectively (Table 5), but no common QTL could be detected in two sub-populations. In ZM-I sub-population, *qPH-6-2_ZM_*/*QPH-6* was the environmentally stable QTL, which was detected in all environments and could explain 14.8 to 34.1% of the phenotypic variation. *qPH-2-1_ZM_*/*QPH-2* and *qPH-4-1_ZM_* were detected in a single environment. In ZM-D sub-population, four QTLs were identified. Their LOD values ranged from 3.2 to 8.7, which explained 9.4 to 35.2% of the phenotypic variation of individual QTL. Among them, *qPH-17-1_ZM_*/*QPH-17* was the most stable QTL detected in all three environments and could explain 9.4 to 30.9% of phenotypic variation. *qPH-10-1_ZM_* and *qPH-13-1_ZM_* were detected in two environments and could explain more than 10% of the phenotypic variation, could also consider as major QTLs for PH in ZM-D population. *qPH-20-1_ZM_* was detected in a single environment. The positive alleles of *qPH-10-1_ZM_* and *qPH-20-1_ZM_* came from *M8206*, while the other five QTLs came from *Zhengyang.*

## 3. Discussion

Plant height (PH) is an important agronomic trait that is closely related to crop yield and quality in soybean [4,12]. Hence, developing soybean cultivars with desirable PH is an important objective of soybean breeders. However, to develop such soybean cultivars, it is imperative to have a detailed understanding of the genetic mechanism as well as genetic elements associated with PH. Plant height is a quantitative trait regulated by multiple genes, and is affected by environmental conditions [26]. Although, over the past decades many QTLs related to soybean PH have been reported, and there are more than 200 QTLs documented for PH in the USDA Soybean Genome Database [13]. However, most of these QTLs were not stable as well as confirmed due to small sized mapping population and low-density genetic map, and hence have not been useful for breeding PH in soybean. The quality of genetic maps has a great influence on the accuracy of QTL detection; high-density genetic map aided in the identification of more recombination events in a population as well as increased QTL mapping accuracy [31,32].

Moreover, little is known about the genetics of PH in summer planting soybean grown in the Chinese Jiang-Huai River Valley [22]. Previous studies have shown that genetic polymorphisms between two soybean types are often very low [33], and QTL detection is often limited in a single mapping population [34]. This suggests that more diverse germplasm are needed for revealing the genetic basis of PH in this specific geographical region. In this regard, the present study utilized high-density linkage maps of two RIL populations viz., MT and ZM derived from diverse parents, and evaluated in multiple environments to identify the main-effect and epistatic effect QTLs as well as their interaction with the environment for PH in soybean. The results of our study showed that there were different QTLs in the two populations and most of QTLs were mapped in a small confidence interval (less than 3 cM), that greatly adds to the growing knowledge of the genetic control of PH (Table 2 and Table 3). 

The QTLs associated with PH has been mapped on all 20 linkage groups/chromosomes of soybean [13]. For cross validation and improving the accuracy of QTL mapping results, we used two different methods for QTL mapping including CIM and MCIM. A total of 8 and 12 additive effect QTLs were detected in MT and ZM populations (including the QTLs were detected in sub-populations), respectively (Table 2, Table 3 and Table 5). Most of QTLs detected in our study overlapped with the loci available on SoyBase database [13], and majority of the previously reported QTLs were identified in small physical genomic regions (Table 2, Table 3 and Table 5), suggesting the importance of using high-resolution genetic map for QTL detection. In particular, the novel QTL, *QPH-17* was identified by comparing the mapping results of both CIM and MCIM methods and in MT and ZM populations (Table 2, Table 3 and Table 5), indicating somehow distinct genetic architecture of MT and ZM populations and suggests the need to use more germplasm for revealing the complex genetic basis of PH in soybean. Finally, a continuous distribution and transgressive segregation were observed for PH in both RIL populations in all environments, which were consistent with the result of previous studies [11,12,22], and implied that the existence of different allelic variations in the loci associated with plant height in the two parents of each RIL population. In fact, the results of this study also show that the positive alleles of the QTLs for PH were derived from both determinate and indeterminate parents in case of both MT and ZM populations. Some lines can pyramid different alleles and the additive effects of the QTLs controlling PH from both parents that make lines with a taller or smaller PH than their parents. Thus, we can develop cultivars with desirable PH by pyramiding different favorable alleles if we know the QTLs associated with PH in summer planting soybean, so it is important to note that not only the higher phenotype parent contributes positive alleles, but also the contribution of positive alleles by lower phenotype parent cannot be disregarded; and similar observation was made by Wang and Guan [35], Miao et al. [36] and Miao et al. [37]. However, the soybean stem growth habit has a great influence on plant height. It is usually better to use the taller determinate cultivars as parents to develop a determinate line with larger plant height, but, we can also use the different QTLs associated with PH that independent of the *Dt1* locus to rapidly develop determinate or indeterminate stem growth habit cultivars. In the present study, the QTLs associated with PH that dependent and independent of the *Dt1* locus were detected. In particular, some QTLs could be detected in different environments and methods. Therefore, our findings provide important information for future soybean breeding programs.

It has been demonstrated that epistatic and QTL by environment interaction effects are the two important genetic factors that makes large contribution to the phenotypic variation observed in complex traits; and the knowledge of those interaction effects are important for understanding the genetic mechanism of complex traits [19,38]. Previous studies revealed that PH of soybean is significantly affected by environment [11,22]. Moreover, knowledge on specific QTL by environment interactions can guide for the search of varieties adapted to particular environments. The QTLs with greater additive effects are often more stable in multiple environments and populations [39,40]. For example, *QPH-19* (additive effect: 12.9) identified in multiple environments and populations; however, *qPH-5-1_ZM_* (additive effect: 2.5) was detected in only one specific environment and population (ZM only) (Table 3). The genetic architecture of PH also includes epistatic interactions between QTLs [22]. Hence, ignoring inter-genic interaction will lead to overestimation of individual QTL effects and underestimation of genetic variance [41]. This in turn could result substantial drop in the genetic response to MAS, particularly at late generations [42]. In this study, six pairs of digeneic epistatic QTLs pairs were identified for PH in both populations, and explained a total of 3.5% and 6.5% of the phenotypic variation in MT and ZM populations, respectively (Table 4). Out of these six pairs, four epistatic QTL pairs did not display additive effects alone, suggesting that these loci might serve as modifying genes that interact with other genes to affect the phenotype of plant height. All six pairs have significant AA but only two QTL pairs viz., *qPH-6-2_MT_* & *qPH-19-1_MT_*/*QPH-19* and *qPH-2-2_ZM_* & *qPH-4-1_ZM_* possess significant AAE interaction effects. However, the total phenotypic variation explained by six epistatic pairs through AA effects was about 10%, and by AAE was 2.7%. Together, the effects of epistatic and environmental interactions (including AA, AE and AAE) could explain 13.9% and 14.7% of phenotypic variation in MT and ZM populations, respectively. Therefore, the results showed that epistatic and environmental interactions are important for understanding genetic basis of PH in soybean, suggesting that these effects should be considered in QTL mapping program, and will increase the accuracy of phenotypic value prediction in MAS. 

In plant breeding, stability of QTL is essential for their use in marker-assisted breeding. Four pairs of QTLs viz., *qPH-2-1_MT_* & *qPH-2-1_ZM_* (*QPH-2*), *qPH-6-3_MT_* & *qPH-6-2_ZM_* (*QPH-6*), *qPH-17-1_MT_* & *qPH-17-1_ZM_* (*QPH-17*) and *qPH-19-1_MT_* & *qPH-19-1_ZM_* (*QPH-19*) were detected in different populations in the same or overlapping physical position, could be considered the same locus. Out of these stable QTLs, *QPH-6* and *QPH-19* have *R*^2^ > 10%, and were considered as the major and stable QTLs. However, *QPH-6* and *QPH-19* were identified in the physical interval that overlap with the previously identified QTLs *viz*., *Plant height 20-3* and *Plant height1-1*, respectively as reported earlier by Gai et al. [43] and Mansur et al. [44]. The *QPH-17* was detected at the physical position of about 40–42 Mb and did not overlap with any of the previously reported PH QTLs, therefore, this stable QTL is been reported for the first time. The *QPH-2* was identified in the physical interval that overlaps with the previously identified QTLs *Plant height 13-1* [45]. In addition to *QPH-2*, *QPH-6*, *QPH-17* and *QPH-19*, some QTLs were identified in a single population through both mapping methods as well as multiple environments. For instance, *qPH-6-2_MT_* was identified in MT population in three environments and overlap with the *Plant height 13-2* [45]. The QTLs viz., *qPH-9-1_ZM_* and *qPH-10-1_ZM_* were detected in two environments in ZM population, were adjacent or overlapping with QTLs as reported by Kim et al. [46] and Wang et al. [6]. Therefore, our results showed the reliability of QTL mapping in the present study, those QTLs can be used as important targets to identify the candidate genes and MAS in future studies.

It is of great interest for both theoretical study and practical breeding program to identify the actual candidate gene underlying the QTL region. Most of the earlier QTL mapping studies on PH did not mine for candidate genes [11,22,26], and till date only few/limited genes related to PH have been isolated from soybean [27,29,30]. Hence, based on the available information in current literature and gene annotation, the present study predicted some of the possible candidate genes for PH that underlies the major and stable QTLs in this study. For example, *Dt1* (*Glyma19g22160*) gene lying within the physical genomic interval of *QPH-19* was predicted the candidate gene. Previous study has revealed that *Dt1* controls soybean growth habits with a significant impact on PH [29]. In addition to the growth habit genes, the maturity and flowering time genes also have a great impact on plant height [22,47,48,49]. The physical intervals of *qPH-6-2_MT_*, *qPH-10-1_ZM_* and *qPH-16-1_ZM_* contain *E1* [48], *E2* [50] and *E9* [51] gene, respectively, and these genes might be the important candidate genes for these QTLs. However, the PH in soybean is known to be controlled by multiple genes. Some genes which have functions related to growth hormones or have functions that are directly or indirectly related to vegetative growth in stable QTLs were predicted as possible candidate genes. A total of 84 and 125 model genes were mined from the physical regions of the two other stable QTLs viz., *QPH-6* and *QPH-17*, and out of these 10 and 9 were considered as possible candidate genes based on the gene annotation and available literature (Table S2). However, it needs further verification and functional validation to prove their actual role in the regulation of PH in soybean. 

## 4. Materials and Methods

### 4.1. Plant Materials and Field Experiments

In the present study, we used two related recombinant inbred line (RIL) populations developed from the *M8206* × *Tongshan* (MT) and *Zhengyang* × *M8206* (ZM) crosses sharing a common parent (*M8206*), and consist of 289 and 126 RILs, respectively. The common parent, *M8206* has a indeterminate stem growth habit, whereas *Zhengyang* and *Tongshan* have determinate stem growth habit. These two RIL populations were derived by advancing F_2_ lines of both populations up to seven generations through single seed descent (SSD) method. Both RIL populations along with their parents were grown in three different environments during normal summer growing season (from June to October) viz., Jiangpu Experiment Station, Nanjing, Jiangsu Province, in year 2012 (2012JP); Jiangpu Experiment Station, Nanjing, Jiangsu Province, in year 2014 (2014JP); and Yancheng Experiment Station, Yancheng, Jiangsu Province, in year 2014 (2014YC). These two populations were planted in a randomized complete block design (RCBD) with one row per plot, 1.0 m row length, 0.5 m row spacing and 0.1 m plant spacing, and three replications were used in each environment. Standard cultural and agronomic practices were followed in each environment. 

### 4.2. Phenotypic Data Analysis 

Plant height (PH) was measured in centimeters (cm) by randomly selected three individual plants in the middle of each row at maturity stage. The PH was defined as the length between the cotyledon node and the peak of main stem. In each individual environment, the PH for each line used for analysis was an average over the three individual plants across three replications.

Descriptive statistics such as mean, standard deviation (SD), range, skewness, kurtosis and coefficient of variation (CV%) for PH were calculated using the SPSS Statistics 20.0 (SPSS Inc., Chicago, IL, USA). The analysis of variance (ANOVA) was carried out by using the SAS PROC GLM program (SAS Institute, 2010. SAS/STAT software version 9.2. SAS Institute Inc, Cary, NC). The broad-sense heritability (*h*^2^) for PH of both RIL populations was estimated using the following equation:

*h*^2^=*σ*^2^_g_/(*σ*^2^_g_+*σ*^2^_ge_ /*n*+*σ*^2^_e_/*nr*)

where *σ*^2^_g_, σ^2^_ge_, σ^2^_e_, *n* and *r* represents genotypic variance, genotype-by-environment interaction variance, error variance, number of environments and number of replications, respectively [52].

### 4.3. QTL Analysis

In the present study, two intra-specific high-density bin maps of two RIL populations viz., MT and ZM earlier developed and published by Li et al. [53], were used for QTL mapping. Both high-density bin maps covered all 20 linkage groups (LGs) and was constructed using bin markers of each RIL population through JoinMap 4.0 software [54]. Genetic distance of each LG is expressed in centiMorgan (cM), and was calculated from recombination frequencies using Kosambi mapping function [55]. The high-density genetic linkage maps for MT and ZM populations contain 3598 and 2600 bin markers, respectively. The total length of the MT and ZM maps were 2451.7 cM and 2630.0 cM with average distance between markers were 0.7 cM and 1.0 cM, respectively. Average length of each LG was 122.6 cM and 131.5 cM for MT and ZM linkage maps with the mean marker number of each linkage group was 130.0 and 179.9, respectively (Supplementary Table S3). 

Composite interval mapping (CIM) method in the Windows QTL Cartographer 2.5 software [56] was employed to detect the additive effect QTLs in each environment to evaluate the environmental stability of the additive effect QTL. For CIM method, the window size, working speed, control marker number and permutation times were set at 10 cM, 1 cM, 5 cM and 1000, respectively, in all three different environments. Significance value (α) of 0.05 was used to determine treatment differences. QTLs identified in different environments but are located at the same, adjacent, or overlapping marker intervals were considered the same QTL. MapChart 2.1 software [57], was used to draw linkage map of both RIL population for locating the position of QTLs on each chromosome/linkage group.

Mixed-model based composite interval mapping (MCIM) method in QTL Network version 2.2 software was used to analyze QTL genetic-effects including additive (A), additive by additive (AA, epistatic) effects and their environmental interaction effects, i.e., additive by environment interaction effects (AE), and epistatic by environment interaction effects (AAE) [58]. The *F*-value threshold was calculated with 1000 permutation tests for the MCIM method. Markov Chain Monte Carlo (MCMC) method was used to estimate QTL effects with 20,000 Gibbs sampler iterations and candidate interval selection and putative QTL detection, and the QTL effects were calculated with an experiment-wise type I error under α= 0.001.

However, the soybean growth habit gene, *Dt1* has a great effect on plant height. Therefore, we also divided the MT and ZM population into two sub-populations, i.e., the determinate stem growth habit sub-population (*dt1dt1*) and the indeterminate stem growth habit sub-population (*Dt1Dt1*), according to the genotype of each line at *Dt1* locus. Finally, in the MT population, there are 114 lines in the determinate stem growth habit (MT-D) population, and 175 lines in the indeterminate stem growth habit (MT-I) population. For the ZM population, there are 51 lines in the determinate stem growth habit (ZM-D) population, and 75 lines in the indeterminate stem growth habit (ZM-I) population. And then, the sub-populations were used to detect the QTLs that independent of the *Dt1* locus by the CIM method in three environments.

The QTLs were named following the popular nomenclature suggested by McCouchet al. [59] with minor modifications. For instance, in case of QTL named *qPH-1-1_ZM_*, *q* represents the QTL, *PH* means plant height, −*1* represents Chromosome 1, −*1* represents its order on the chromosome, and *_ZM_* means this QTL was detected in ZM population. Similar naming was followed for other QTLs identified in the present study.

## 5. Conclusions

In conclusion, the present study was the detailed investigation for elucidating the genetic architecture of PH in Chinese summer planting soybean, in which we used high-density genetic maps of two RIL populations (MT and ZM) to identify major and stable QTLs associated with PH. In total, we identified eight and 12 additive effect QTLs in MT and ZM populations (including the QTLs were detected in sub-populations), respectively. Among all QTLs, four QTLs viz., *QPH-2*, *QPH-6*, *QPH-17* and *QPH-19* were consistently identified in two populations and/or multiple environments and mapping methods, hence, these QTLs regions will be the major focus of soybean breeders for fine mapping and marker-assisted breeding of ideal PH in summer planting soybean. Based on physical genomic position, three QTLs viz., *qPH-4-2_ZM_*, *qPH-15-1_MT_* and *QPH-17* were novel, out of which *QPH-17* were identified as stable QTL. Furthermore, six QTLs showed significant AE interaction effects in both populations, and also six digenic epistatic QTLs pairs were identified. Our findings might be of great usefulness for marker-assisted breeding, and could provide detailed information for accurate QTL localization and gene discovery.

## Figures and Tables

**Table 1:** Plant height (cm) observed in MT and ZM populations and their parents.

Population	Environment	Parents			RILs						
		*M8206*	*Tongshan*	*Zhengyang*	Mean	SD	Range	Skewness	Kurtosis	CV (%)	*h*^2^ (%)
MT	2012JP	62.1 ± 3.1	73.3 ± 8.3	-	67.6	18.0	19.5–126.8	0.4	0.4	26.6	92.3
	2014JP	67.8 ± 7.4	75.4 ± 11.1	-	89.0	26.2	17.8–163.1	0.1	0.2	29.5	
	2014YC	58.2 ± 8.4	75.0 ± 5.0	-	76.3	15.6	22.3–112.3	−0.9	2.0	20.4	
ZM	2012JP	62.1 ± 3.1	-	76.2 ± 11.1	80.9	19.0	34.0–130.3	0.1	−0.4	23.5	92.5
	2014JP	67.8 ± 7.4	-	75.7 ± 6.7	93.4	21.3	43.0–167.1	0.7	1.0	22.8	
	2014YC	58.2 ± 8.4	-	77.7 ± 13.7	71.7	11.5	42.3–101.3	0.4	0.2	16.0	

Where RILs, SD, CV, JP (2012JP and 2014JP) and 2014YC represents recombinant inbred lines, standard deviation, coefficient of variation, Jiangpu experiment station (in 2012 and 2014) and Yancheng experiment station, respectively.

**Table 2:** Detection of QTL associated with plant height in two populations across the three different environments by CIM method.

Population	QTL ^a^	Chr.	Position (cM)	Marker Interval	LOD ^b^	Confidence interval (cM) ^c^	Physical Region (Mb) ^d^	A ^e^	R^2^ (%) ^f^	Env.	ReportedQTL/Gene ^g^
MT	*qPH-2-1_MT_* (*QPH-2*)	2	19.3	T_bin192–T_bin193	3.8	18.1–21.5	4.5–5.2	−2.7	3.0	2014YC	*Plant height 13-1*
	*qPH-6-2_MT_*	6	82.8	T_bin992–T_bin993	28.6	82.6–83.0	19.1–21.9	−9.8	29.3	2014YC	*E1*
			83.0	T_bin994–T_bin995	26.9	82.7–83.2		−13.9	19.4	2014JP	
			83.0	T_bin994–T_bin995	14.1	82.7–83.3		−7.8	13.7	2012JP	
	*qPH-6-3_MT_*	6	87.1	T_bin1020–T_bin1021	26.4	87.0–87.4	42.9–45.4	−12.7	19.2	2014JP	*Plant height 3-2*
	(*QPH-6*)		89.8	T_bin1028–T_bin1029	13.3	89.6–92.7		−7.0	13.0	2012JP	
	*qPH-15-1_MT_*	15	60.7	T_bin2638–T_bin2639	3.2	59.3–61.0	14.4–15.4	3.6	1.8	2014JP	Novel
	*qPH-16-1_MT_*	16	67.9	T_bin2836–T_bin2837	3.4	66.7–68.5	30.3–30.8	−2.5	2.7	2014JP	*E9*
	*qPH-19-1_MT_*	19	97.7	T_bin3421–T_bin3422	23.8	97.0–98.7	44.9–45.5	7.6	22.2	2014YC	*Dt1*
	(*QPH-19*)		97.3	T_bin3420–T_bin3421	55.7	96.5–97.8		19.4	50.7	2014JP	
			97.3	T_bin3420–T_bin3421	31.2	96.5–97.9		11.0	33.9	2012JP	
ZM	*qPH-5-1_ZM_*	5	51.4	Z_bin554–Z_bin555	2.9	48.9–53.4	32.1–32.9	−2.9	6.1	2014JP	*Plant height 26-1*
	*qPH-6-2_ZM_*	6	117.7	Z_bin718–Z_bin719	6.5	117.3–118.7	37.8–43.9	−7.1	11.3	2012JP	*Plant height 3-2*
	(*QPH-6*)		118.6	Z_bin719–Z_bin720	7.5	117.3–122.8		−8.6	12.7	2014JP	
	*qPH-7-1_ZM_*	7	100.2	Z_bin879–Z_bin880	3.0	98.6–101.0	38.0–38.6	3.0	6.5	2014YC	*Plant height 37-5*
	*qPH-9-1_ZM_*	9	110.6	Z_bin1195–Z_bin1196	4.6	109.7–111.5	40.7–42.4	−5.3	7.6	2012JP	*Plant height 33-5*
			108.6	Z_bin1192–Z_bin1193	2.8	106.6–111.9		−4.3	3.9	2014JP	
	*qPH-10-1_ZM_*	10	104.2	Z_bin1330–Z_bin1331	3.4	102.7–106.8	43.6–44.4	4.8	5.5	2012JP	*E2*
			104.2	Z_bin1330–Z_bin1331	4.5	101.7–105.1		6.1	7.2	2014JP	
	*qPH-17-1_ZM_* (*QPH-17*)	17	134.1	Z_bin2179–Z_bin2180	2.7	126.0–135.1	40.3–41.9	−4.1	4.2	2012JP	Novel
			134.1	Z_bin2179–Z_bin2180	5.1	132.7–135.1		−6.4	8.2	2014JP	
			127.7	Z_bin2175–Z_bin2176	3.8	126.9–128.6		−3.5	9.0	2014YC	
	*qPH-18-1_ZM_*	18	88.3	Z_bin2283–Z_bin2284	4.0	85.1–91.0	53.5–54.6	3.6	9.3	2014YC	*Dt2*
	*qPH-19-1_ZM_*	19	108.8	Z_bin2431–Z_bin2432	15.0	108.4–109.5	44.6–45.9	11.4	30.9	2012JP	*Dt1*
	(*QPH-19*)		108.8	Z_bin2431–Z_bin2432	10.8	108.2–110.1		10.2	19.7	2014JP	

^a^ QTLs detected in different environments at the same; adjacent or overlapping marker intervals were considered the same QTL. ^b^ LOD value at the peak > 2.5. ^c^ 1-LOD support confidence intervals (confidence interval length, cM). ^d^ physical location of the confidence interval of QTLs in the *Glycine max* Wm82.a1 reference genome (Mb). ^e^ The additive effect of QTLs. The positive values indicated that the allele of the QTL came from the common parent *M8206* can increase plant height. While negative the values indicate that the allele of the QTL came from other parents that can increase plant height. ^f^ Phenotypic variance (%) explained by the QTL. ^g^ The name of QTL for plant height in Soybase [13] or the gene name which associated with plant height within the QTL interval.

**Table 3:** Additive QTL and interaction effect between QTL and environment for plant height detected by MCIM method in the two populations.

Population	QTL	Chr.	Marker Interval	Position (cM)	Confidence Interval (cM)	A ^a^	*R*^2^ (A) (%) ^b^	AE ^c^	*R*^2^ (AE) (%) ^d^	Physical Interval (Mb)	ReportedQTL/Gene ^e^
MT	*qPH-6-2_MT_*	6	T_bin992–T_bin993	82.8	82.6–83.1	−7.5 **	13.5	−2.5 ** (AE2)/3.1 ** (AE3)	1.9	19.1–21.3	*E1*
	*qPH-6-3_MT_* (*QPH-6*)	6	T_bin1028–T_bin1029	89.8	89.6–91.0	−3.7 **	3.2			44.1–45.0	*Plant height 20-3*
	*qPH-16-1_MT_*	16	T_bin2833–T_bin2834	63.9	61.3–66.8	−3.0 **	2.1			29.5–30.3	*E9*
	*qPH-17-1_MT_*(*QPH-17*)	17	T_bin3042–T_bin3043	116.3	116.1–116.3	−2.7 **	1.7			40.8–41.8	Novel
	*qPH-19-1_MT_* (*QPH-19*)	19	T_bin3419–T_bin3420	96.4	96.3–97.7	12.9 **	39.3	−3.4 ** (AE1)/6.3 ** (AE2)/−2.7 ** (AE3)	6.9	44.7–45.3	*Dt1*
ZM	*qPH-5-1_ZM_*	5	Z_bin554–Z_bin555	53.4	49.9–55.6	−2.6 **	2.1			32.1–33.3	*Plant height 26-1*
	*qPH-6-2_ZM_*	6	Z_bin723–Z_bin724	123.1	122.0–124.4	−4.8 **	7.2	−1.8 * (AE2)	0.9	43.8–44.23	*Plant height 20-3*
	*qPH-10-1_ZM_*	10	Z_bin1334–Z_bin1335	106.4	103.3–106.8	4.9 **	7.6	−1.8 * (AE3)	1.0	43.8–44.4	*E2*
	*qPH-17-1_ZM_*(*QPH-17*)	17	Z_bin2179–Z_bin2180	134.1	132.7–135.5	−4.9 **	7.6	−1.7 * (AE2)	0.7	41.0–41.9	Novel
	*qPH-18-1_ZM_*	18	Z_bin2283–Z_bin2284	87.3	86.7–90.3	4.2 **	5.5			53.5–54.5	*Dt2*
	*qPH-19-1_ZM_* (*QPH-19*)	19	Z_bin2434–Z_bin2435	111.5	109.8–111.6	6.3 **	12.5	−4.3 ** (AE1)/2.0 * (AE2)/2.4 * (AE3)	4.5	44.8–45.9	*Dt1*

* indicates *p <* 0.05; **indicates *p <* 0.01. ^a^ additive effect: positive value indicate that *M8206* contributed the allele for an increase in the trait value. ^b^ phenotypic variance explained by additive QTL. ^c^ additive QTL by environment interaction effects. AE1 indicates 2012JP; AE2 indicates 2014JP; AE3 indicates 2014YC. ^d^ phenotypic variance explained by additive QTL with environment interaction effect. ^e^ The name of QTL for plant height in Soybase [13] or the gene name which associated with plant height within the QTL interval.

**Table 4:** Epistatic effects (AA) and environmental (AAE) interaction of QTLs for plant height in two populations.

Population	Pair	QTL	Chr.	Position (cM)	Marker Interval	Confidence Interval (cM)	Physical Interval (Mb)	AA ^a^	*R*^2^ (AA) (%) ^b^	AAE ^c^	*R*^2^ (AAE) (%) ^d^
MT	*1*	*qPH-6-2_MT_*	6	82.8	T_bin992–T_bin993	82.6–83.1	19.1–21.3	1.1 *	0.3	2.7 ** (AE1)/	1.6
		*qPH-19-1_MT_*	19	96.4	T_bin3419–T_bin3420	96.3–97.7	44.7–45.3			−2.4 ** (AE3)	
	2	*qPH-1-1_MT_*	1	49.8	T_bin89–T_bin90	46.6–49.9	42.5–46.0	−2.6 **	1.6		
		*qPH-6-1_MT_*	6	69.7	T_bin953–T_bin954	68.8–72.2	14.9–15.6				
	3	*qPH-9-1_MT_*	9	115.8	T_bin1696–T_bin1697	113.6–116.7	45.3–46.2	−2.6 **	1.6		
		*qPH-20-1_MT_*	20	91.9	T_bin3581–T_bin3582	89.5–96.2	42.0–43.9				
ZM	*1*	*qPH-5-1_ZM_*	5	53.4	Z_bin554–Z_bin555	49.9–55.6	32.1–33.3	−2.3 **	1.6		
		*qPH-17-1_ZM_*	17	134.1	Z_bin2179–Z_bin2180	132.7–135.5	41.0–41.9				
	2	*qPH-1-1_ZM_*	1	28.3	Z_bin22–Z_bin23	24.1–31.6	3.2–4.4	−2.2 **	1.5		
		*qPH-6-1_ZM_*	6	108.5	Z_bin701–Z_bin702	106.0–111.2	16.2–18.0				
	3	*qPH-2-* *2_ZM_*	2	49.5	Z_bin165–Z_bin166	48.6–50.2	6.8–8.2	−3.3 **	3.4	−2.2 * (AE3)	1.1
		*qPH-4-1_ZM_*	4	61.7	Z_bin433–Z_bin434	60.3–65.6	8.7–11.0				

* indicates *p <* 0.05; ** indicates *p <* 0.01. ^a^ epistatic effects, a positive value indicates that the parental two-locus genotypes have a positive effect and that the recombinants have a negative effect. ^b^ phenotypic variance explained by epistatic QTL pair. ^c^ epistatic QTL pairs by environment interaction effects. AE1 indicates 2012JP; AE2 indicates 2014JP; AE3 indicates 2014YC. ^d^ phenotypic variance explained by epistatic QTL pairs with environment interaction effect.

**Table 5:** Detection of QTLs associated with plant height in the sub-populations of MT and ZM populations across the three different environments by CIM method.

Sub-Populations ^a^	QTL	Chr.	Position (cM) ^b^	Flanking Marker	LOD	Physical Interval (Mb)	Additive Effect	R^2^ (%)	Env.	Reported QTL/Gene ^c^
MT-D	*qPH-6-2_MT_*	6	82.8	T_bin992-T_bin993	22.2	18.8–21.9	−15.4	49.6	2014YC	*E1*
			82.8	T_bin992-T_bin993	15.8		−14.3	35.7	2014JP	
			83.1	T_bin994-T_bin995	6.5		−6.9	16.7	2012JP	
	*qPH-16-1_MT_*	16	67.9	T_bin2836-T_bin2837	3.1	29.0–31.0	−3.5	7.2	2012JP	*E9*
			68.5	T_bin2837-T_bin2838	4.6		−4.2	6.2	2014YC	
			69.5	T_bin2840-T_bin2841	4.2		−5.0	7.4	2014JP	
MT-I	*qPH-6-2_MT_*	6	83.1	T_bin994-T_bin995	13.7	19.3–21.9	−6.4	21.4	2014YC	*E1*
			83.0	T_bin993-T_bin994	15.5		−12.5	28.8	2014JP	
	*qPH-6-3_MT_*	6	86.9	T_bin1016-T_bin1018	13.6	41.3–43.8	−6.2	21.3	2014YC	*Plant height 20-3*
	(*QPH-6*)		87.5	T_bin1022-T_bin1023	15.8		−12.1	29.2	2014JP	
			87.5	T_bin1022-T_bin1023	9.4		−7.4	18.3	2012JP	
	*qPH-15-2_MT_*	15	74.3	T_bin2691-T_bin2692	5.4	43.5–47.0	3.6	7.8	2014YC	*Plant height* *26-10*
	*qPH-17-1_MT_* (*QPH-17*)	17	114.9	T_bin3040-T_bin3041	3.4	40.3–41.8	−4.2	5.9	2012JP	Novel
ZM-I	*qPH-2-1_ZM_* (*QPH-2*)	2	30.0	Z_bin145-Z_bin146	8.3	4.6–5.2	−5.7	23.5	2014YC	*Plant height* *13-1*
	*qPH-4-* *2_ZM_*	4	74.1	Z_bin451-Z_bin452	3.8	37.0–40.7	−5.4	11.0	2012JP	Novel
	*qPH-6-2_ZM_*	6	125.3	Z_bin726-Z_bin727	10.7	37.8–45.2	−12.8	34.1	2014JP	*Plant height 20-3*
	(*QPH-6*)		121.1	Z_bin721-Z_bin722	7.3		−5.6	22.2	2014YC	
			118.6	Z_bin725-Z_bin726	5.0		−6.4	14.8	2012JP	
ZM-D	*qPH-10-1_ZM_*	10	95.3	Z_bin1320-Z_bin1321	8.7	42.2–43.8	10.7	35.2	2012JP	*E2*
			98.6	Z_bin1325-Z_bin1326	4.2		6.4	13.5	2014JP	
	*qPH-13-1_ZM_*	13	42.4	Z_bin1598-Z_bin1599	3.2	20.3–21.4	−6.4	13.8	2014JP	*Plant height* *37-8*
			42.4	Z_bin1598-Z_bin1599	4.0		−6.7	14.1	2012JP	
	*qPH-17-1_ZM_*	17	134.1	Z_bin2179-Z_bin2180	6.5	40.2–41.9	−9.4	24.0	2014JP	Novel
	(*QPH-17*)		128.1	Z_bin2176-Z_bin2177	8.2		−6.5	30.9	2014YC	
			134.1	Z_bin2179-Z_bin2180	3.2		−6.0	9.4	2012JP	
	*qPH-20-1_ZM_*	20	51.7	Z_bin2531-Z_bin2532	3.8	35.5–37.2	4.1	11.5	2014YC	*Plant height* *16-1*

^a^ MT-D and ZM-D represent the sub-populations composed of lines with determinate stem growth habit in MT and ZM populations, respectively. MT-I and ZM-I represent the sub-populations composed of lines with indeterminate stem growth habit in MT and ZM populations, respectively. ^b^ The position of the QTL peak marker on the original map of the MT and ZM populations. ^c^ The name of QTL for plant height in Soybase [13] or the gene name which associated with plant height within the QTL interval.

## Supplementary Materials

The following are available online at https://www.mdpi.com/2223-7747/8/10/373/s1, Table S1: Analysis of variance (ANOVA) of plant height in MT and ZM populations over three environments, Table S2: The candidate genes related with soybean plant height in stable QTL intervals, Table S3: Summary genetic maps in ZM and MT populations.

## Abbreviations

PH	plant height
QTL	quantitative trait loci
RIL	recombinant inbred line
CIM	composite interval mapping
MCIM	mixed-model based composite interval mapping
MAS	marker-assisted selection
SNP	single nucleotide polymorphism
JP	Jiangpu experiment station
YC	Yancheng experiment station
SD	standard deviation
CV	coefficient of variation
MS	mean square
cM	centiMorgan
A	additive
AA	additive by additive interaction
AE	additive by environment interaction
AAE	epistatic by environment interaction
