# Supplementary material for: Deciphering the Genetic Architecture of Plant Height in Soybean Using Two RIL Populations Sharing a Common M8206 Parent
Source: Plants (Basel). 2019 Sep 26;8(10):373. doi: 10.3390/plants8100373 (PMC6843848; doi:10.3390/plants8100373)
Supplement: Supplementary file 1 [file plants-08-00373-s001.pdf]

Supplementary materials

Table S1 Analysis of variance (ANOVA) of plant height in MT and ZM populations over three environments.

Population	Source	DF[a]	MS[b]	*F*[c]
MT	Genotype	288	2858.19	12.97**
	Environment	2	101597.76	461.15**
	Block within Environment	6	6579.24	29.86**
	Genotype × environment	573	360.94	1.64**
	Error	1651	220.31	
ZM	Genotype	125	2118.32	13.34**
	Environment	2	44416.89	279.66**
	Block within Environment	6	2009.65	12.65**
	Genotype × environment	250	346.91	2.18**
	Error	741	158.82	

**$P < 0.01$

[a] Degrees of freedom

[b] Mean square

[c] *F* value is used to determine significance

Table S2 The candidate genes related with soybean plant height in stable QTL intervals

QTL	Gene name[a]	Function annotation
qPH-6	*Glyma06g40700*、*Glyma06g40730*、*Glyma06g40750*、*Glyma06g40810*	response to auxin stimulus
	Glyma06g40800	glycolysis
	Glyma06g40860	auxin homeostasis, response to auxin stimulus, auxin mediated signaling pathway
	Glyma06g41520	gibberellin biosynthetic process, gibberellic acid mediated signaling pathway
	Glyma06g41530	cell differentiation
	Glyma06g41600	cytokinesis by cell plate formation
	Glyma06g41072	auxin polar transport
qPH-17	*Glyma17g37090*	determination of bilateral symmetry, regulation of meristem growth
	Glyma17g37120	response to auxin stimulus, response to ethylene stimulus, positive regulation of auxin metabolic process
	Glyma17g37370	positive regulation of auxin metabolic process, regulation of meristem growth, auxin polar transport, multidimensional cell growth
	Glyma17g37650	tissue development
	Glyma17g37760	cell tip growth, response to gibberellin stimulus
	Glyma17g37810	response to ethylene stimulus
	Glyma17g38000	regulation of unidimensional cell growth
	Glyma17g38150	gibberellic acid mediated signaling pathway
	Glyma17g38210	cell proliferation

[a]The gene were annotated in Wm82.a1 reference genome.

Table S3 Summary genetic maps in ZM and MT populations

Population	Chromosome	Bin numbers	Genetic distance of bin (cM)	
			Mean	Total
ZM	Gm01	117	1.05	122.1
	Gm02	144	1.11	158.7
	Gm03	117	0.82	94.8
	Gm04	120	1.07	127.1
	Gm05	109	0.92	99.3
	Gm06	158	1.06	166.6
	Gm07	135	0.93	124.1
	Gm08	185	1.02	187.3
	Gm09	131	0.98	128
	Gm10	137	0.98	133.3
	Gm11	123	1.17	143.2
	Gm12	83	1.26	103.3
	Gm13	158	1.14	178.5
	Gm14	106	1.11	116
	Gm15	126	0.98	123.1
	Gm16	113	1.04	116.4
	Gm17	119	1.15	135.9
	Gm18	165	0.79	130.4
	Gm19	114	1.18	133.9
	Gm20	140	0.78	108.4
	**Total**	**2600**	**1.01**	**2630.2**
MT	Gm01	157	0.63	102.4
	Gm02	174	0.84	148.5
	Gm03	200	0.55	109.8
	Gm04	171	0.7	119.1
	Gm05	152	0.74	112
	Gm06	223	0.54	123
	Gm07	200	0.63	125.7
	Gm08	224	0.78	174.6
	Gm09	195	0.6	119.1
	Gm10	159	0.87	137.8
	Gm11	154	0.91	145.8
	Gm12	134	0.74	102.3
	Gm13	225	0.59	140.9
	Gm14	168	0.65	112.7
	Gm15	173	0.59	101.7
	Gm16	167	0.57	96.4
	Gm17	153	0.77	116.5
	Gm18	226	0.49	114.7
	Gm19	183	0.65	140.4
	Gm20	160	0.66	108.2
	**Total**	**3598**	**0.68**	**2451.7**
